# Energy landscapes of planar colloidal clusters

**DOI:** 10.1039/c4nr02670e

**Published:** 2014-08-06

**Authors:** John W. R. Morgan, David J. Wales

**Affiliations:** a University Chemical Laboratories , Lensfield Road , Cambridge CB2 1EW , UK; b University Chemical Laboratories , Lensfield Road , Cambridge CB2 1EW , UK . Email: dw34@cam.ac.uk

## Abstract

A short-ranged pairwise Morse potential is used to model colloidal clusters with planar morphologies. Potential and free energy global minima as well as rearrangement paths, obtained by basin-hopping global optimisation and discrete path sampling, are characterised. The potential and free energy landscapes are visualised using disconnectivity graphs. The short-ranged potential is found to favour close-packed structures, with the potential energy primarily controlled by the number of nearest neighbour contacts. In the case of quasi-degeneracy the free energy global minimum may differ from the potential energy global minimum. This difference is due to symmetry effects, which result in a higher entropy for structures with lower symmetry.

## Abstract 2

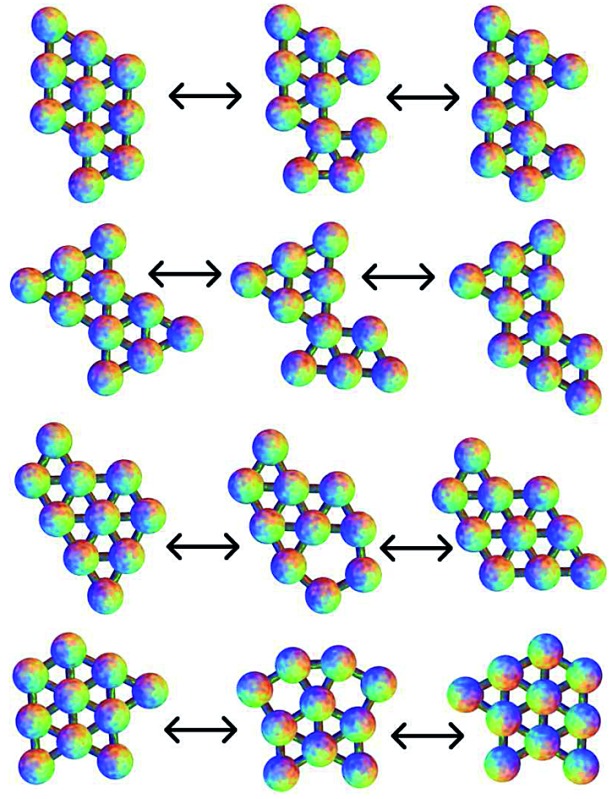
A short-ranged pairwise Morse potential is used to model colloidal clusters with planar morphologies. Low-lying potential energy minima, free energy minima and rearrangement mechanisms states are predicted.

## 1. Introduction

The synthesis of colloidal particles has progressed rapidly, moving beyond simple isotropic particles.^1–9^ However, there is still much work to be done theoretically to understand small clusters of colloids.^10^ Here we focus on models of two-dimensional planar colloidal clusters, representing systems deposited on a solid surface, or at a liquid–liquid or liquid–gas interface.^11,12^ Since such particles can be viewed by optical microscopy, the systems are open to both theoretical modelling and experimental testing.^13^ Two-dimensional systems have thus far received less theoretical attention, but are well characterised experimentally under various conditions.^14–16^ Theory and models help to explain the behaviour observed in experiments and can highlight interesting avenues for experimental investigation.

The Morse potential^17^ has been used extensively in previous work to model the properties of various atomic and molecular clusters.^18–25^ The range of the Morse potential can be adjusted, with a long range (relative to the particle radius) being appropriate for atomic clusters, such as sodium, and a much shorter range for clusters of fullerene molecules.^22^ In the present work, colloidal particles are modelled by coarse-graining: real colloidal particles correspond to the nanometre to micrometre length scale and consist of thousands of atoms or molecules, but the effective inter-particle potential is usually represented by a single isotropic site.^26^ Colloid particles have an even shorter range effective interaction than fullerenes, for example corresponding to about 1.05 times the particle radius,^13^ as a result of the larger excluded volume.

The interaction between particles is assumed to be pairwise additive, and this simple assumption has been successful in predicting properties of colloids observed by optical microscopy.^27^ While other experiments have shown that there can be significant deviation from pairwise additivity in some circumstances, this deviation occurs when there are longer-range interactions present, for example when the electrostatic screening length is longer than the inter-particle separation.^28^ Here, we use a short-ranged potential, so the deviation from pairwise additivity is expected to be small. The short range of the potential has two significant effects on the energy landscape. First, it favours close-packed structures,^29–33^ since deviations from the equilibrium distance between pairs will lead to a larger strain energy than with a longer-range interaction. The higher strain contribution arises because the energy increases more rapidly with distance for a short-ranged potential compared to a long-ranged potential. Second, a short-ranged potential has more local minima and corresponds to a potential energy landscape that is globally smooth but locally rough, an effect we have explained using catastrophe theory.^34,35^

In the present work, we present an analysis of low-lying minima and the pathways between them for planar Morse clusters consisting of between six and ten particles. We also include some results for the three-dimensional Morse cluster of thirteen particles for comparison. The thirteen particle cluster was chosen since it is small enough to permit definitive computations, and is large enough to exhibit a variety of structures.

## 2. Methods

### 2.1 The Morse Potential

The interaction between two colloid particles is modelled using the Morse potential,^17^ written as

**Fig. 1:**
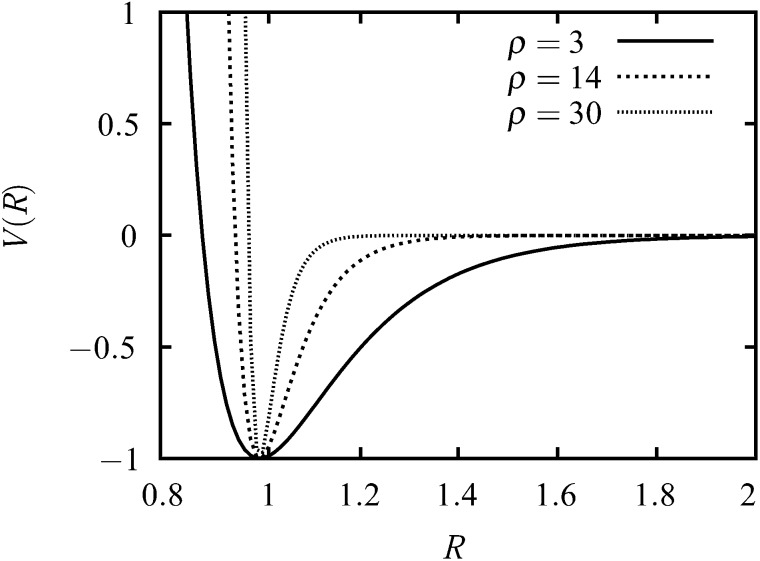
Plots of the Morse potential with *ρ* = 3, *ρ* = 14 and *ρ* = 30. The distance is in units of *R*_e_ and the energy is in units of *ε*.

1 *V* = *ε*e^*ρ*(1–*R*/*R*_e_)^(e^*ρ*(1–*R*/*R*_e_)^ – 2),

where *R*_e_ is the equilibrium distance between the particles and *ε* is the energy at *R* = *R*_e_, so that *ρ* is a parameter that controls the range of the potential.^17^ The longer-range Morse potential, with *ρ* ≈ 3, has been used to study sodium clusters, while a shorter range, with *ρ* ≈ 14 is more appropriate for clusters of C_60_.^22^ In the present work, *ρ* ≈ 30 is used, as for previous work on colloidal particles.^10,27,36,37^ The total energy for the clusters is simply the sum of all the pair energies. Plots of the potential for several values of *ρ* are shown in Fig. 1.

### 2.2 Global optimisation

The global potential energy minimum and other local minima were identified by basin-hopping (BH) constrained to a plane^38–41^ using the GMIN program^42^ including some additional seeded searches. Up to 10^5^ BH steps were run in an attempt to ensure that all minima had been located. Evaporation of particles from the cluster was prevented using a method based on maintaining the connectivity of the graph formed by considering each particle as a vertex and each inter-particle distance of less than 1.2 *R*_e_ as an edge.^43^ The BH steps were taken as random Cartesian displacements.

### 2.3 Transition state searches

The doubly-nudged^44,45^ elastic band^46–48^ method, as implemented in the OPTIM program,^49^ was used to identify likely candidates for transition states between selected pairs of minima. The candidates were then tightly converged to structures with a single negative Hessian eigenvalue, according to the Murrell–Laidler definition of a transition state,^50^ using a hybrid eigenvector-following approach.^51^ The approximate steepest-descent paths corresponding to the positive and negative directions of the unique Hessian eigenvector were followed to find the minima that each transition state connected.

All minima and transition states were converged to a tolerance of 10^–12^ for the root mean square gradient. This value was chosen to ensure the differentiation of minima that are very close in energy. The PATHSAMPLE program^52^ was used to expand the database of minima and transition states^53,54^ to the point where all minima and pathways connecting each pair had probably been found. The databases were visualised using disconnectivity graphs.^55,56^ All the planar stationary points had three zero eigenvalues, corresponding to two orthogonal translations within the plane, and rotation about the axis perpendicular to the plane. ‘Floppy’ structures, with additional zero eigenvalues,^35,57,58^ were not considered.

### 2.4 Thermodynamics

Once a database of local minima has been found, thermodynamic properties can be calculated approximately using the harmonic superposition approach, in which the global partition function is assumed to be the sum of the harmonic partition functions of the individual basins.^59–66^ This calculation requires the selection of a temperature of interest, which we took as *k*_B_ *T*/*ε* = 0.25 in line with experimental conditions and previous work.^10,13^ The partition function for each minimum was approximated by assuming a harmonic vibrational potential and ignoring translational and rotational contributions,^65^ which can be shown to have a negligible effect.

Permutation isomers of each structure are grouped together, increasing the thermodynamic weight of each structure. The number of permutation-inversion isomers of a structure α is given by

2 *n*_α_ = *N*!/*o*_α_,

where *N* is the size of the cluster (since all particles are identical) and *o*_α_ is the order of the point group of α.^65,67^ For a two-dimensional structure, inversion is equivalent to a two-fold rotation about the axis perpendicular to the plane, and need not be considered separately.

The thermodynamic weight is reduced for structures with higher point group symmetry, while according to the principle of maximum symmetry, the lowest lying minima on the potential energy landscape are likely to have higher symmetry.^68,69^ The partition function for structure α is given by3
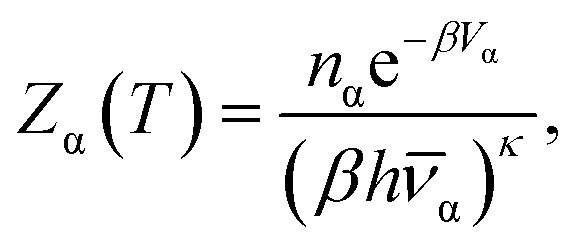
where *β* = 1/*k*_B_ *T*, *V*_α_ is the potential energy of α, *ν̄*_α_ is the geometric mean of the vibrational normal mode frequencies, and *κ* = 2*N* – 3, the number of two-dimensional vibrational degrees of freedom.^65^ The total partition function of the system is obtained by summing the partition functions for the individual structures. This representation is explicitly ergodic.

Particles interacting *via* short-ranged potentials, with locally rough but globally smooth landscapes, are expected to have multiple structures of similar energy to the global minimum, so the symmetry of the cluster is expected to play a crucial role in determining the global free energy minimum.^13,27,70^

### 2.5 Kinetics

Once the pathway between two minima is known, forward and backward rates can be calculated using transition state theory,^71–76^ employing a harmonic density of states consistent with the harmonic superposition approach, combined with a graph transformation procedure.^77–80^ Where the pathway includes more than one transition state, the lowest energy pathway can be constructed from multiple minimum-transition state-minimum paths using the missing-connection algorithm.^45^ All these procedures are implemented in our PATHSAMPLE program.

### 2.6 Point group symmetry

To describe clusters confined to a plane we consider two-dimensional point groups. The possibilities are cyclic groups *C*_*n*_, consisting of the *n* rotations for a *C*_*n*_ axis and therefore having order *n*; and the pyramidal groups *C*_*n*v_, consisting of the *n* rotations for a *C*_*n*_ axis, as well as the reflections in *n* planes including that axis, with order 2*n*. The group containing just the identity element is *C*_1_ and that containing the identity and one mirror plane is *C*_s_ (which is equivalent to *C*_1v_).

## 3. Results

### 3.1 The six-particle cluster

**Fig. 2:**
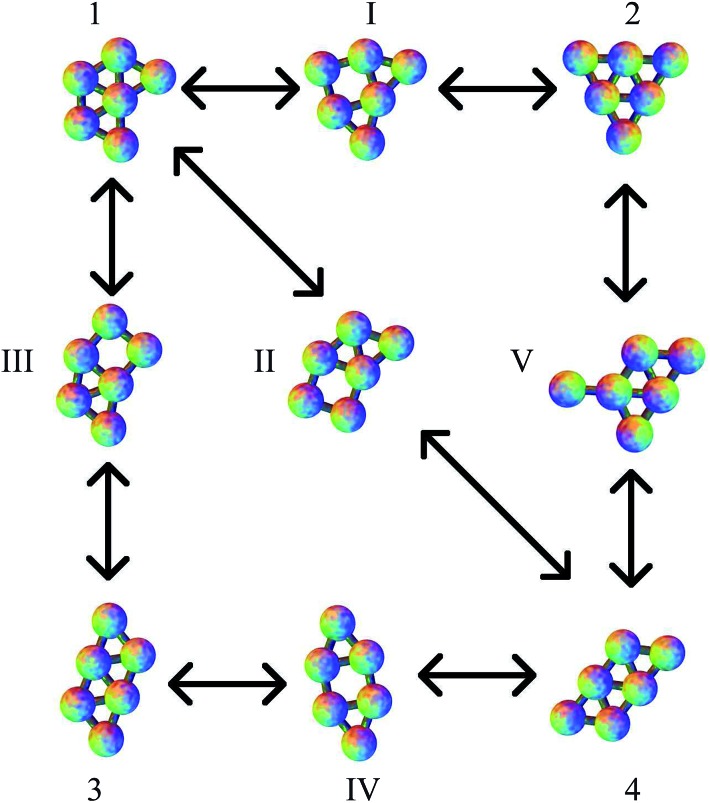
The four minima for the six particle cluster, with transition states connecting them. Except for transition state V, these pathways correspond to the DSD mechanism. The numbering of minima and transition states is used consistently, with Arabic numerals used for minima and Roman numerals used for transition states. Edges are for illustration only, and indicate a pair of particles close to having the equilibrium separation.

The planar Morse cluster with six particles has only four local minima. Eight transition states were found. The energies and symmetries of the minima and transition states are collected in Table 1. The structures of the minima and five of the transition states are shown in Fig. 2. Throughout, minima are numbered by Arabic numerals and transition states by Roman numerals. Images were produced using the POV-Ray ray-tracer.^81^ Minimum 1 is the global potential energy minimum. Both minima 1 and 2 have a mirror plane perpendicular to the plane of the molecule, so they are not chiral. Minima 3 and 4 do not have any two-dimensional symmetry elements other than the identity, and are enantiomers, even though they would correspond to the same structure if they were permitted to rotate out-of-plane. Transition states II and III, which connect minima 3 and 4 to minimum 1, are therefore necessarily also enantiomers, as are transition states V and VI, which connect minima 3 and 4 to minimum 2.

**Table 1:** The energies, free energies (*k*_B_ *T*/*ε* = 0.25) and point groups of the minima and transition states for the six-particle cluster. Energies are in units of *ε*

Structure	Energy	Free Energy	Group
1	–9.000000002319481	0	*C*_s_
2	–9.000000001739894	0.2788907667	*C*_3v_
3	–9.000000001739707	0.01615146697	*C*_2_
4	–9.000000001739707	0.01615146697	*C*_2_
I	–8.000016045019636		*C*_s_
II	–8.000016045596524		*C*_1_
III	–8.000016045596524		*C*_1_
IV	–8.000016045018192		*C*_2v_
V	–8.000000002319483		*C*_1_
VI	–8.000000002319483		*C*_1_
VII	–8.000016046268708		*C*_s_
VIII	–8.000016045688762		*C*_s_

**Fig. 3:**
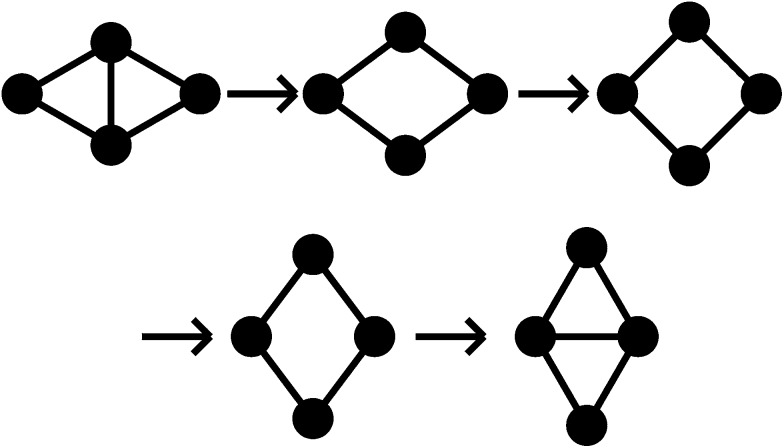
A schematic illustration of the diamond-square-diamond pathway. Circles represent particles and lines connect particles with nearest-neighbour contacts.

All pathways for transition states I to IV correspond to the diamond-square-diamond (DSD) process^82^ previously characterised for three-dimensional short-ranged Morse clusters, as well as for atomic clusters, such as boranes.^10^ This mechanism is illustrated in Fig. 3. From the initial arrangement of two equilateral triangles, the top and bottom particles move up and the side particles move inwards, until they reach a favourable nearest-neighbour separation. The interparticle distances are maintained around the edges of the transition state, so only one nearest-neighbour contact is broken during the process.

**Fig. 4:**
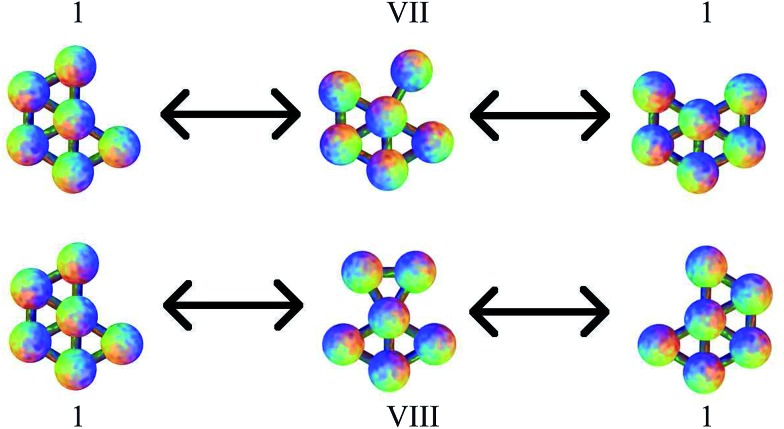
Transition states VII and VIII, connecting different permutational isomers of minimum 1. These pathways do not correspond to a DSD process.

Four more transition states were found, between permutational isomers of minimum 1, and between minimum 2 and minima 3 and 4, where the mechanism is different (Fig. 4). Transition states V, VI and VII represent the rotation of one particle about the central atom, while transition state VIII represents the concerted rotation of two particles about the central atom.

**Fig. 5:**
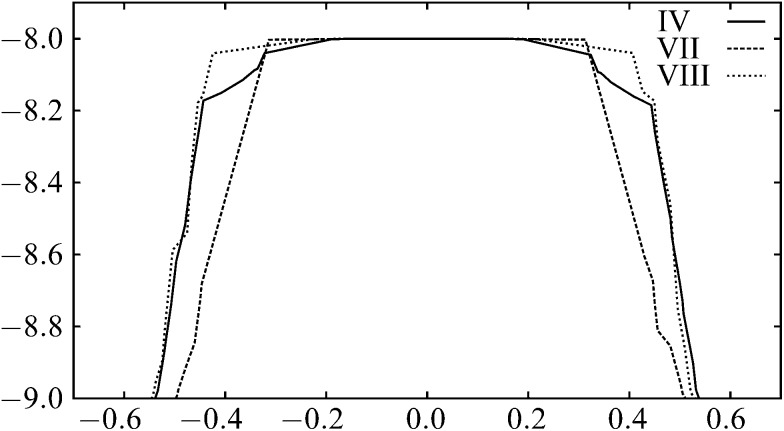
The variation of energy along the integrated path length for the paths including transition states IV, VII and VIII in the six-particle cluster.

To compare the different mechanisms, the plots of the energy along the integrated path length for the rearrangements including transition states IV, VII and VIII are shown in Fig. 5; the energies are clearly very similar. For longer-range potentials, the partial bonding across the centre of the square in the transition state for a DSD pathway reduces the energy and makes the path more favourable. With this short-ranged potential, the bonding interactions across the square are negligible, as we see from the energies of the transition states in Table 1. Since each pathway involves the breaking of one nearest-neighbour contact, and then the formation of another, the energetics of the pathways are similar.

The pathways all appear ‘flat’ around the transition state. This effect is again due to the short range of the potential.^83^ The energy rises quickly as the nearest-neighbour contact is broken. As the structure moves through the transition state, no contacts are being made or broken, so the profile is relatively flat.

**Fig. 6:**
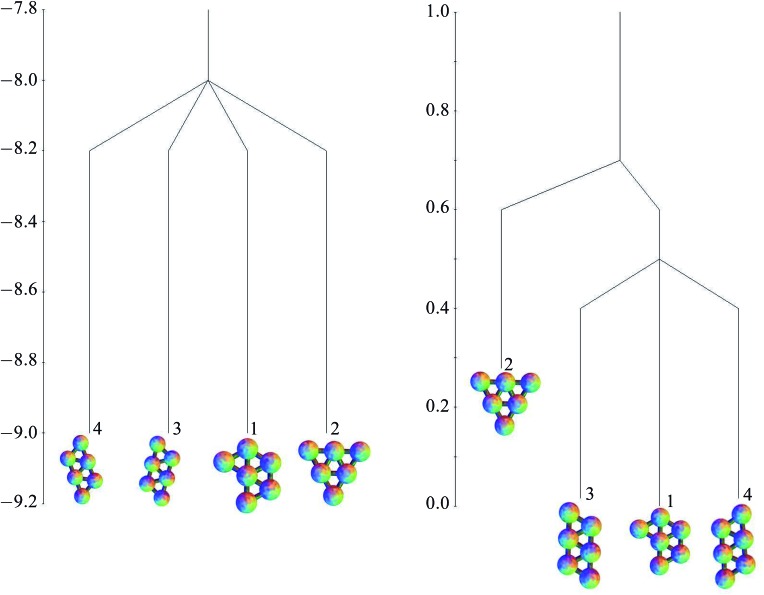
Potential energy (left) and free energy (right) disconnectivity graphs (*k*_B_ *T*/*ε* = 0.25) for the six-particle cluster.

The free energies at *k*_B_ *T*/*ε* = 0.25, based on the harmonic superposition approximation as described in Section 2.4, are also shown in Table 1. The free energy zero is taken to be the free energy of the global minimum. The potential energies of the minima are all very similar, but for the free energies, the effect of symmetry is apparent. Minima 1, 3 and 4, with a point group order of 2, have comparable free energies, but minimum 2, with a point group order of 6, lies higher. The potential and free energy landscapes are summarised in the disconnectivity graphs in Fig. 6.

### 3.2 Global minima of larger clusters

Databases of minima and transition states were also constructed for clusters of 7, 8, 9 and 10 particles. The number of structures in each database are shown in Table 2, increasing approximately exponentially with system size. The number of minima approximately doubles at each successive size, while the number of transition states increases by about a factor of three.^62,84^

**Table 2:** Database sizes for the different cluster sizes considered

Cluster Size	Number of minima	Number of transition states
6	4	8
7	6	18
8	14	59
9	27	171
10	62	535

The energies of the global potential energy minima are shown in Table 3 along with their point group symmetry. Also shown is the quasi-degeneracy of the global minima,^10^ which is the number of strain-free structures with the same number of nearest-neighbour contacts as the global minimum and therefore having very similar energy. In the cases where the global minimum is not quasi-degenerate, there are many quasi-degenerate structures with one fewer nearest-neighbour contact. Quasi-degeneracy is common in the three dimensional Morse clusters for *ρ* = 30,^10^ but we expect it to be less common for the planar systems due to the smaller number of degrees of freedom present. However, to validate this suggestion further, an analysis of many more cluster sizes would be required.

**Table 3:** The global minimum of the potential energy for each cluster, along with the point groups and quasi-degeneracies

Cluster size	Global minimum of potential energy	Point group	Quasi-degeneracy
6	–9.000000002319481	*C*_s_	4
7	–12.000000003479226	*C*_6v_	1
8	–14.000000004059379	*C*_s_	1
9	–16.000000005219306	*C*_s_	5
10	–19.000000006379047	*C*_2v_	1

**Fig. 7:**
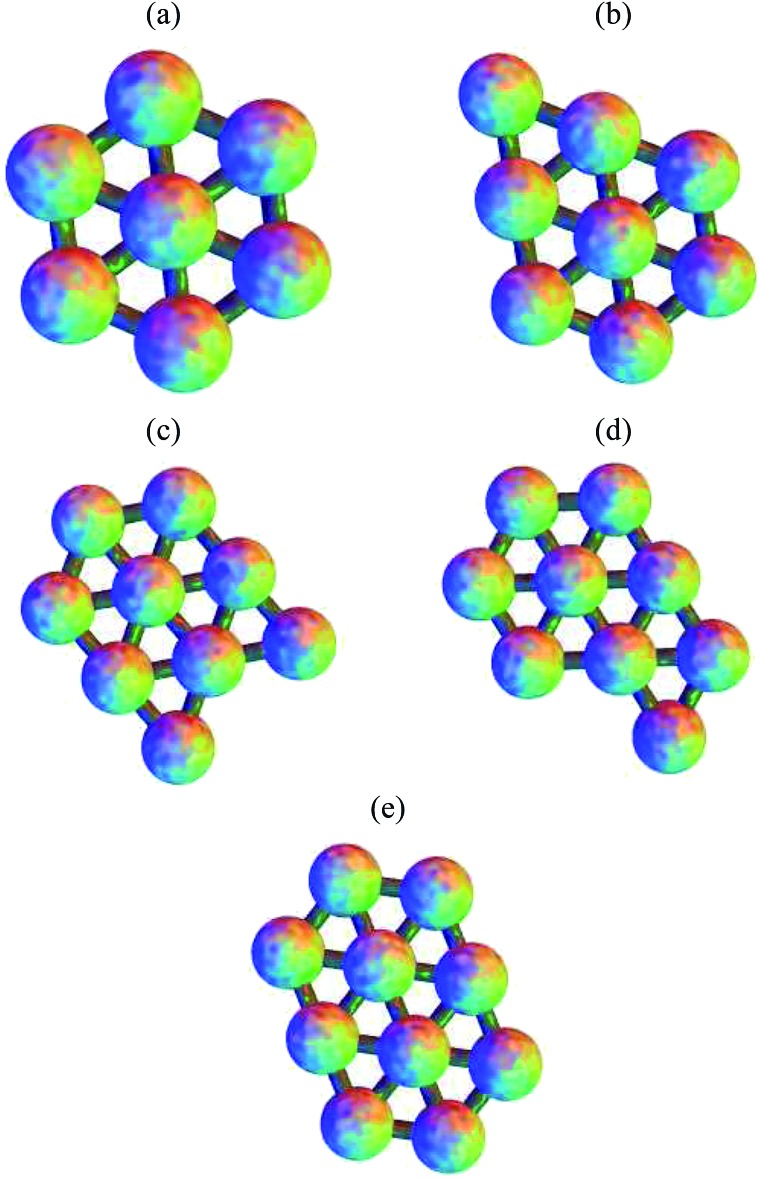
The global potential energy minima for clusters of (a) 7, (b) 8, (c) 9 and (e) 10 particles. (d) The global free energy minimum of the 9-particle cluster.

The structures of the global potential energy minima are shown in Fig. 7. All these minima are fragments of a close-packed hexagonal lattice, which gives the minimal strain energy, as expected for a short-ranged potential.^29–33,83^ For seven particles, the hexagon with a central particle has the most nearest-neighbour contacts. For eight particles, an extra particle can be added on the edge of the hexagon in any one of six equivalent sites. In the nine particle cluster, there are five sites on the edges of the hexagon, and two sites next to the particle outside the hexagon, to which a particle could be added to give a structure with the same number of nearest-neighbour contacts. Hence the quasi-degeneracy of the nine particle cluster is relatively large. Some of the sites are actually equivalent, producing a quasi-degeneracy of five rather than seven. The global minimum is the one where the particle is added on an edge next to the particle outside the hexagon, which has a slightly lower energy. In the ten particle cluster, there is only one site in the nine particle global minimum that gives the maximal number of nearest-neighbour contacts.

For the seven particle, eight particle and ten particle clusters, the global free energy minimum is the same as the global potential energy minimum. Although they all have higher symmetry than the second-lowest potential energy minimum, they have one more nearest-neighbour contact, and the lower potential energy is enough to overcome the increase in free energy due to symmetry at a temperature of *k*_B_ *T*/*ε* = 0.25. However, the quasi-degeneracy of the nine particle cluster means that the free energy global minimum is the one with the lowest symmetry out of the group with the maximal number of nearest-neighbour contacts. This minimum is shown in Fig. 7. Since the order of the point group is one, there are two enantiomers (only one is shown).

### 3.3 Energy landscapes for larger clusters

**Fig. 8:**
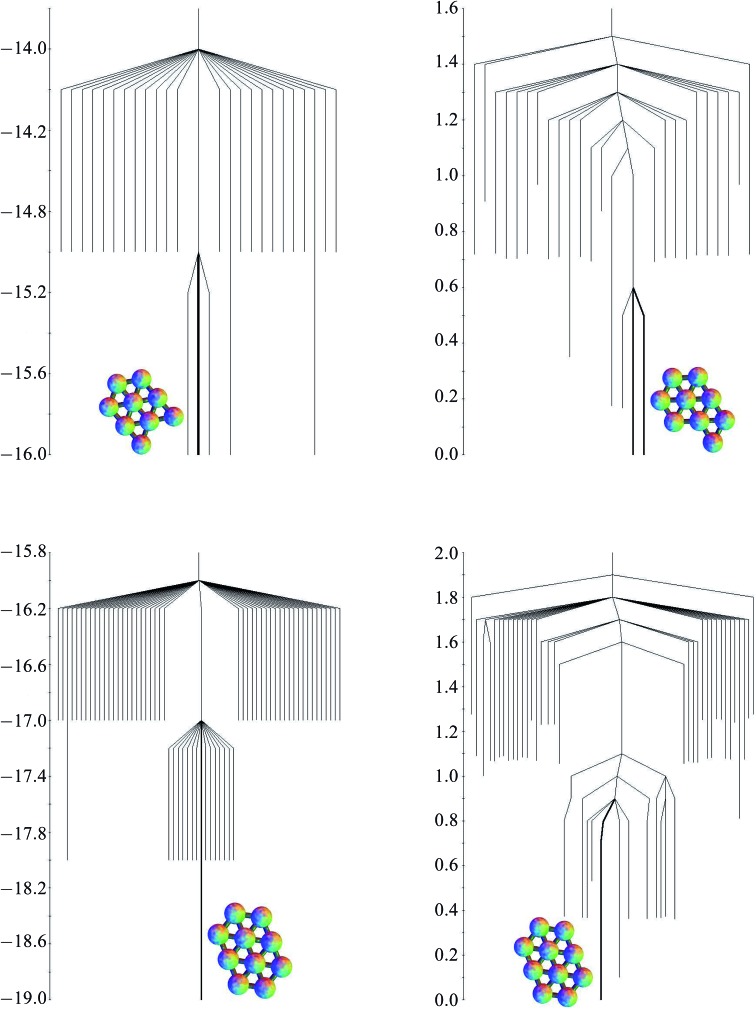
The potential energy (left) and free energy (right) disconnectivity graphs for the nine-particle (top) and ten-particle (bottom) clusters. The branch corresponding to the global minimum is highlighted in bold along with the structure.

As the number of minima and transition states grows, a diagram such as Fig. 2 showing all the minima and the paths between them becomes unwieldy. The potential and free energy landscapes for cluster sizes nine and ten are therefore summarised in the disconnectivity graphs in Fig. 8. The potential energy graphs clearly show the division of minima into groups with the same number of nearest-neighbour contacts and very similar energies. The potential energy graph for the nine particle cluster shows that two of the quasi-degenerate low-lying minima do not lie within the same funnel as the global minimum, suggesting a frustrated landscape,^85,86^ where low-lying structures with different morphologies are separated by high energetic barriers. Comparison with three-dimensional clusters^10^ leads us to expect that larger clusters with quasi-degenerate minima will have frustrated landscapes. In contrast, the ten-particle potential energy graph shows there is no quasi-degeneracy and there is only one minimum with a barrier higher than one energy unit separating it from the global minimum. For both cluster sizes, the free energy graphs show a decrease in the relative barriers between potential kinetic traps and the global minimum.

### 3.4 Transition states for larger clusters

**Fig. 9:**
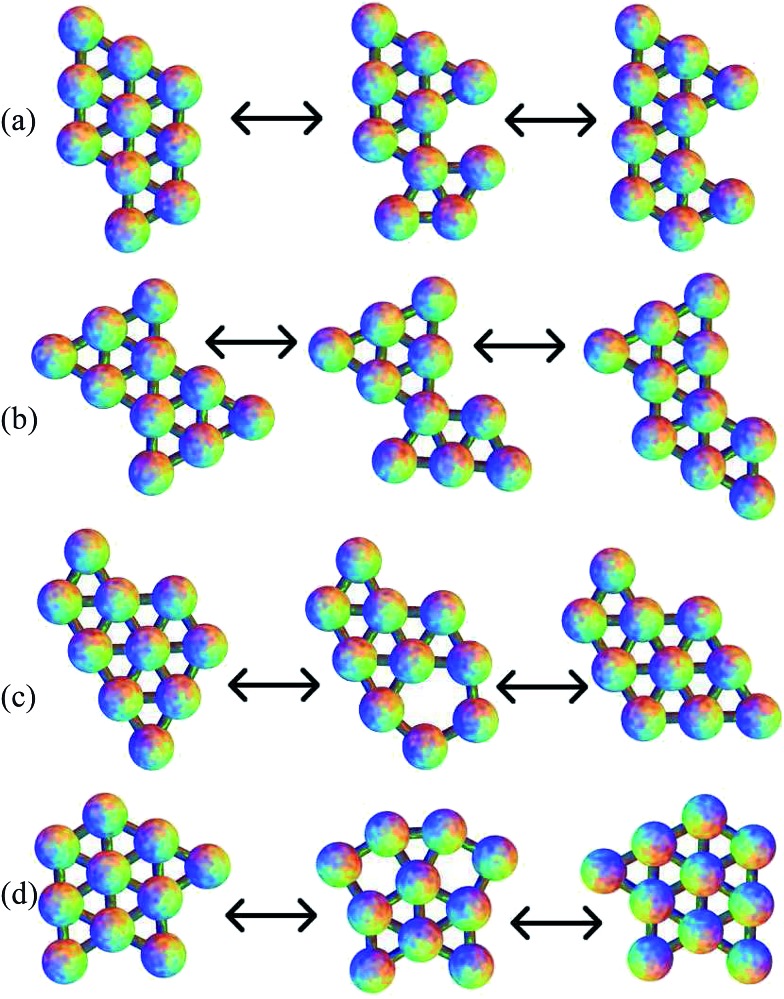
Examples of pathways for the ten-particle cluster showing: (a) rotation of three particles; (b) rotation of four particles; (c) a transition state with a pentagonal ring; and (d) two concerted diamond-square-diamond mechanisms.

Of the 789 transition states located across all the cluster sizes considered, 437 (55%) correspond to a DSD pathway. 93 of the rest follow a pathway similar to transition state VII of the six-particle cluster, with the rotation of one particle on the outside of the cluster around another atom, and 45 are similar to transition state VIII of the six-particle cluster, involving the rotation of two particles that almost maintain a pair equilibrium separation. There are also 38 paths where three particles rotate around another atom in a concerted fashion, and 23 where four particles rotate. 43 of the transition states have a pentagonal arrangement of atoms. The remaining transition states feature two of these mechanisms in a concerted fashion, mostly two concerted DSD rearrangements. The two squares in the transition state can either share an edge, or not. Some of these pathways are illustrated in Fig. 9. In these examples, the rotation of three particles breaks two nearest-neighbour contacts, and then reforms only one, but there are other examples where one contact is broken and another is formed. The rotation of four particles breaks one contact and forms another, but again there are other examples where one contact is broken and two are formed. For the pentagonal and double-DSD pathways, two contacts must always be broken and formed within the squares or pentagon, but other contacts may also be formed or broken during the rearrangements. Some of these pathways are similar to those proposed for boranes and carboranes.^87,88^

### 3.5 Comparison of reaction rates

Rates of reaction between pairs of minima of the ten particle cluster were calculated using PATHSAMPLE and the procedures described in Section 2.5, again at a temperature corresponding to *k*_B_ *T*/*ε* = 0.25. Of the fifteen minima with energy *ε* greater than the global minimum, single step paths to the global minimum have been found for eleven of them. Five follow a DSD pathway with the formation of an extra contact at the end, while the other six follow a double DSD pathway, again with the formation of an extra bond at the end. The rates are collected in Table 4. The first three transitions follow a DSD pathway and involve breaking two bonds and forming one, so the barrier is approximately 2*ε* for the forward reaction (out of the global minimum) and *ε* for the backward reaction. The variation between rates for these three different paths is small and is mostly attributable to the differences in vibrational normal mode frequencies between different structures. The third pathway leads to a minimum with a mirror plane. Since the order of the point group of the starting minimum appears in the numerator of the expression for the rate constant, it is expected that the rate of the backward reaction is greatest for this path. The second set of three rearrangements follow a double-DSD pathway: the forward barrier is approximately 3*ε* and the backward barrier 2*ε*. The reactions are an order of magnitude slower as a result, which suggests that rearrangement pathways breaking the fewest number of nearest-neighbour contacts will be strongly favoured at this temperature.

**Table 4:** Rate constants calculated at *k*_B_ *T*/*ε* = 0.25 for rearrangements of low-lying minima to the global minimum (forward) and from the global minimum to low-lying minima (backward). Only one of each enantiomeric pair is shown, except for the second entry, in which the minimum has a mirror plane and is therefore achiral

Minimum energy	Transition state energy	Forward rate	Backward rate
		× 10^4^	× 10^6^
–18.000000005799084	–17.000022726595279	2.386822684	1.607725433
–18.000000006379050	–17.000022726011633	1.992503156	4.015289903
–18.000000006379054	–17.000022726594068	1.286442635	3.924971563
–18.000000005799269	–16.000039322543525	0.3903622462	0.8928180984
–18.000000005799457	–16.000039321963566	0.3544523320	0.7060362773
–18.000000005799276	–16.000039321960699	0.3526386679	0.7621273829

### 3.6 Comparison with a three-dimensional cluster

Analogous calculations have been applied to the three-dimensional thirteen particle cluster for comparison. The global potential minimum is the *D*_3h_ hexagonal close-packed fragment. The quasi-degeneracy is eight, and the other low-lying structures include the cuboctahedron, less symmetrical close-packed fragments, and a *C*_2v_ structure based on octahedra, which is not close-packed. As for the planar structures, these minima all involve the same number of nearest-neighbour contacts (36 for this cluster) and all have energies only very slightly less than –36*ε*.

If the approximate exponential growth of the number of minima and transitions states were to continue, there would be approximately 450 minima and 15 500 transition states for the planar thirteen particle cluster. For the three-dimensional system, 39 190 minima and 173 038 transition states have been found. This result illustrates the significant increase in complexity when extra degrees of freedom are involved. The planar cluster has 23 degrees of freedom, while the three dimensional cluster has 33.

**Fig. 10:**
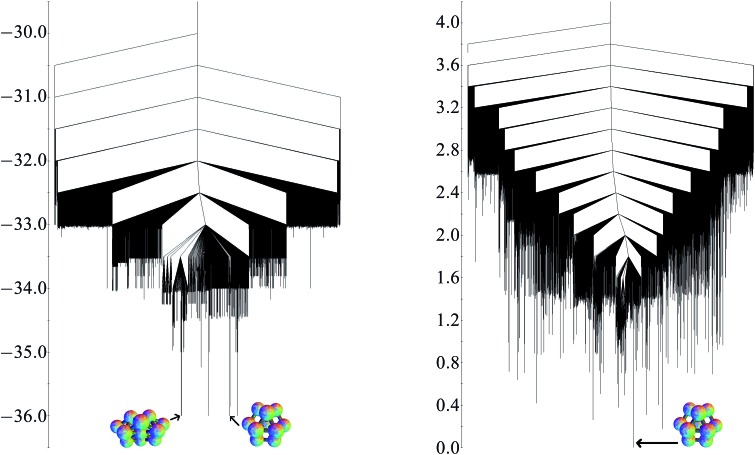
Potential energy (left) and free energy (*k*_B_ *T*/*ε* = 0.25) (right) disconnectivity graphs for the 13-particle three-dimensional cluster. On the potential energy graph, the global minimum *D*_3h_ structure and the quasi-degenerate *C*_2v_ are shown. On the free energy graph, the global minimum *D*_3h_ structure is shown.

The potential energy and free energy disconnectivity graphs are shown in Fig. 10. The potential energy graph clearly illustrates the quasi-degeneracy at all energy levels. There are very few minima with a barrier more than *ε* separating them from the global minimum, creating a funnelled landscape. In contrast, the quasi-degeneracy is lifted in the free energy graph and the landscape appears to be more frustrated.

The pathways corresponding to these transition states generally include concerted motion of more particles than for the planar clusters. However, analogous processes involving the rotation of one particle or three particles and the DSD rearrangement have also been identified. These pathways are similar to those previously described for the 19-particle cluster.^10^

## 4. Conclusions

The present work examines the structure, dynamics and thermodynamics of small planar colloidal clusters. We have characterised the global and other low-lying potential energy minima for cluster sizes between 6 and 10 particles, identified by basin-hopping. From this starting point, we have calculated free energy minima for the relevant experimental conditions and described the most important transition states and pathways between minima.

These data have been used to provide some more general observations about the factors influencing structural stability: the potential energy is largely controlled by the number of nearest-neighbour contacts and therefore close-packed structures are favoured.^29–33^ The symmetry is important for determining the free energy minimum when quasi-degeneracy is present.^10^ The majority of the rearrangements correspond to a diamond-square-diamond process, but there are also pathways involving the migration of a single particle or small groups of particles around the edge of the cluster.

Our predictions can be tested against experimental observations for colloids deposited on surfaces using optical microscopy to check the applicability of the simple Morse potential employed here. Analysis of rearrangement mechanisms and kinetics should prove to be particularly interesting for these intriguing systems. Furthermore, the databases of minima and transition states may be useful for benchmarking and testing other structure prediction methods.
